# Principles of Human Physiology, with Their Chief Applications to Psychology, Pathology, Therapeutics, Hygiene, and Forensic Medicine

**Published:** 1853-04

**Authors:** 


### REVIEWS.

---

ART. III.—*Principles of Human Physiology, with their chief applications to Psychology, Pathology, Therapeutics, Hygiene, and Forensic Medicine.* By WILLIAM B. CARPENTER, M. D., F. R. S., F. G. S., Examiner in Physiology and Comparative Anatomy in the University of London, Professor of Medical Jurisprudence in University College, etc. Fifth American from the fourth and enlarged London edition, with three hundred and fourteen illustrations. Edited, with additions, by FRANCIS GURNEY SMITH, M. D., Professor of the Institutes of Medicine in the Medical Department of Pennsylvania College, Lecturer on Physiology in the Philadelphia Association for Medical Instruction, etc. Philadelphia, Blanchard & Lee, 1853: 8 vo., pp. 1091.

Few works have obtained, in this country, greater popularity than the above treatise of Dr Carpenter, and the present edition will fully sustain its high reputation as one of the clearest, most philosophical, and most practical works extant on the subject. It affords precisely what the student of physiology wants, a faithful view of that science as it now stands. The author has so far modified and enlarged the present edition, that it may be considered in the light of a new work, "the old material having been incorporated with the new, rather than the new with the old." In order that the reader may see how far it has been reconstructed, we copy from his preface an outline of the changes which have been made:

Considering it extremely important that his readers should have a clear idea of the sense in which the terms *law* and *cause* are subequently employed, he has devoted a few pages of the *Introduction* to an explanation of his views upon these points, and he hopes that he may be there found to have thrown some light upon the philosophy of causation, which may be of assistance to other scientific inquirers.

In order to make room for a portion of the new matter which he desired to introduce into the treatise, he has felt it necessary to omit all those references to the structure and vital actions of the lower animals which had not an immediate and direct bearing upon human physiology; and consequently, of the first chapter of the previous editions—"On the Place of Man in the Scale of Being"—he has only retained so much as related to the characteristics that distinguish man from the mammalia which most nearly approach him. The succeeding chapter, which treated "Of the Different Branches of the Human Family, and their Mutual Relations," has been extended in all that relates to man, and curtailed in that which rather belongs to Comparative Physiology, and has been transferred to nearly the end of the volume, which the author considers to be now the more appropriate place for it.

The second chapter of the present edition, comprising a general view "Of the Chemical Components of the Human Body, and the Changes which they undergo within it," is now for the first time introduced. Several new views will be found in this chapter, which have occurred to the author during its preparation; he would especially point to that of the respective relations of fibrine and albumen to the nutritive processes, and of the former to the gelatinous tissues; and to the general summary which forms the last section, in which the discoveries of M. Cl. Bernard, in regard to the elaboration of sugar and fat in the liver, are placed, he believes, in a somewhat novel aspect.

From the consideration of the chemical components of the organism, and of the participation of chemical forces in its operations, it seemed natural to pass on to that of "The Structural Elements of the Human Body, and the Vital Actions which they Exhibit,' which forms the subject of the third chapter. Nearly the whole of this chapter, which includes the general doctrines of cell-formation and of vital force, in their application to human physiology, appears for the first time, in this edition.

Passing on to the more detailed survey of the constituent parts of the human body, the first place seemed to be claimed by the b.ood, the "physical characters, chemical composition, and vital properties of which are treated of at some length in Chapter IV. This portion has been greatly extended, and almost entirely re-written; the great importance of the subject, in its bearings on Pathology as on Physiology, having been constantly kept in view.

The fifth chapter, "On the Primary Tissues of the Living Body, their Structure, Composition, and Action," is essentially the same with the third chapter of the previous edition; but a large amount of new matter, in great part supplied by the elaborate "Mikroscopische Anatomie," of Professor Kölliker, has been incorporated in it; and many new illustrations, chiefly derived from the same source, have been introduced. The account of the vital endowments of the muscular and nervous tissues, previously contained in other chapters, has been transferred to this, so as to make it embody a complete sketch of those physiological actions of these separate parts of the organism, which are afterwards to be considered in their relations to each other.

In conformity with the opinion expressed by some of his friendly critics, and by many teachers of Physiology, the author has reversed the previous arrangement of the chapters which treat of the functions in detail; those relating to the organic functions being now placed *before* those in which the animal functions are described instead of *after*. This has involved a new distribution of much of the matter which was previously treated in a connected form in the chapter on the "Functions of the Nervous System," since it has appeared to the author very desirable that the whole group of actions whose aggregate makes up each function, should now be considered in its connexion, and thus the movements of deglutition, respiration, etc., not having been explained, as was formerly the case, in the earlier part of the volume, are described, and their connexion with the nervous system examined, under each separate head. As their general relations to the nervous system are previously explained, however, in the sixth chapter, the author does not apprehend that any inconvenience will be experienced from this alteration.

The series of chapters on the several organic functions remain essentially the same as in the previous edition; but important additions and corrections have been made in every one. Thus, in Chapter VII., "On Food and the Digestive Process," the whole subject of Food is much more fully discussed than heretofore; and the most important of the results obtained from the study of the digestive process by Frerichs, Bernard, and other experimenters, have been embodied in the account of it. In Chapter VIII., "On Absorption and Sanguification," the structure and development of the Ductless Glands have been more fully described, in accordance with the researches of Kölliker, Sanders, Ecker, Gray, and others, and their relation to the process of sanguification more clearly elucidated. In Chapter IX., "On the Circulation of the Blood," the causes of the heart's sounds have been more fully considered; a view of the nature of its rhythmical contractions has been suggested, which the author believes to be original; and the most important among the results of Prof. Volkmann's elaborate researches on the dynamics of the movement of the blood have been introduced. In Chapter X., "On Respiration," the most important additions to the first section are those which embody the results of Dr. Hutchison's inquiries on the movements of respiration; to the second the data furnished by the researches of MM. Regnault and Reiset, Prof. Scharling, M. Barral, and others, upon the amount of oxygen absorbed, and of carbonic acid exhaled; whilst the third, in which the "Effects of Suspension or Deficiency of Respiration" are discussed, has been largely augmented by a summary of the evidence afforded by our recent experience, of the marked tendency of an habitually imperfect respiration to produce a liability to zymotic disease. Nearly the whole of Chapter XI., "On Nutrition," has been newly written for this edition. In Chapter

XII., "On Secretion and Excretion," important additions have been made under almost every head; and those parts, especially, which relate to the agency of the excretory apparatus in maintaining the purity of the blood, have been extended. This chapter, however, is less comprehensive than formerly; several of the subjects which it previously included having been tranferred to portions of the work in which they seemed to find more appropriate places; the salivary and pancreatic secretions being now treated of in the chapter on Digestion, and those of the testes and mammæ in that on Generation. Of the three subjects included in Chapter XIII., "On the Evolution of Heat, Light, and Electricity," the first alone had been systematically considered in the previous editions, and this has been considerably extended in the present. Under the second head will be found some very curious observations on the evolution of light in the living human subject; and under the third is given a summary of the admirable researches of M. Du Bois-Raymond, which have been recently brought before the scientific public in this country by Dr. Bence Jones.

It is in the chapter (XIV.) devoted to the Functions of the Nervous System, which constitutes one-fifth of the entire volume, that the greatest additions and alterations will be found. This subject, in its Psychological as well as in its Physiological relations, has occupied more of the author's attention than any other department of Physiology; and he now offers the more matured fruits of his inquiries and reflections, with some confidence that, even if his views should hereafter require modification as to its details, they will be found to be fundamentally correct, and to furnish materials of some value in Psychological inquiry, as well as in the study of Mental Pathology. * * The peculiar states which are known under the names of somnambulism, hypnotism, mesmerism, electro-biology, etc., are all considered in their relations to sleep on the one hand, and to the ordinary condition of mental activity on the other; and the author ventures to believe that he has not only succeeded in throwing considerable light upon the nature of these aberrant forms of psychical action, but that he has been enabled to deduce from their phenomena some inferences of great importance in Psychological science.

In Chapter XV., "On Sensation, and the Organs of the Senses," comparatively little change has been made; several additions have been introduced, however, and some corrections made. The next chapter (XVI.), "On Muscular Movements," has been entirely remodelled; the portion which relates to the vital endowments of muscular fibre having been removed to Chapter V., Section 6, and its place supplied by new matter, which contains many original views, especially under Section 4, which treats of the "Influence of Expectant Attention on Muscular Movements." Comparatively little alteration has been found necessary in Chapter XVII., "On the Voice and Speech," or in Chapter XVIII., "On the Influence of the Nervous System on the Organic Functions;" an important addition has been made to the latter, however, with reference to the influence of the state of "expectant attention" on the operations of nutrition, secretion, etc.

The additions and alterations which have been made in Chapter XIX, "On Generation," will be found to be both numerous and important, especially under the section on the "Development of the Embryo," which has been almost entirely re-written, so as to bring the view of this process more into accordance with the existing state of our knowledge of it. The author has not felt it expedient, however, to enter into minute details upon this subject.

In Chapter XX., "On the Different Branches of the Human Family, and their Mutual Relations," all that directly relates to this subject has been considerably extended, and many novelties have been introduced; whilst those arguments for the specific unity of the human races which are derived from the analogy of the lower animals, have been simply referred to, having been fully dwelt on by the author elsewhere.

The closing chapter, "On Death," has been almost entirely written for this edition, the subject having been only touched on incidentally in the preceding.

Of the manner in which Dr. Carpenter applies physiology to the illustration of pathology we have an admirable example in the second chapter of his work. It has reference to diabetes, which was long regarded as a disease of the kidneys. Bernard has shown that sugar is a normal constituent of the liver; that it is found in that organ whether the food of the animal be farinaceous or saccharine, or not; that it is susceptible of being formed out of the protien compounds. If from any cause an excess of sugar be generated, it must appear in the urine, the combustive process not being adequate to its consumption. Hence, we are to look rather to the liver than the kidneys for the origin of diabetic sugar. In the liver of a diabetic patient, Bernard found no less than 833 grains of sugar. The liver, in this disease, seems to exercise its metamorphic power upon the azotized constituents of the blood, and thus to destroy the material for the nutritive processes. This affords an explanation of the rapid wasting in diabetic subjects, notwithstanding that the appetite may be good, and large quanties of food taken.

This agency of the liver in converting all articles of food into sugar, appears to be influenced by the conditions of the nervous system. When the upper portion of the medulla oblongata is irritated, the production of sugar is increased to a great extent; an artificial diabetes is induced, which continues for a short time. This change in the character of the urine has been brought about in twenty minutes by this operation. If the lesion be too great, instead of an increase in the saccharine secretion, it is entirely suspended.

In a healthy state of the system the sugar developed is converted into lactic acid, and is eliminated by the lungs, an evolution of heat being the result. But notwithstanding the great amount of sugar, and its speedy conversion into carbonic acid, in the experiments of Bernard it was found that the temperature of the animals rapidly declined. This last fact, our author remarks, appears to stand in marked opposition to the chemical theory of animal heat; but he proposes the following explanation which removes the difficulty:

The production of heat being dependent upon the combustion (or union with oxygen), not merely of carbon, but of hydrogen, and the amount of heat disengaged by the combustion of hydrogen being much greater than that given off by the combustion of its equivalent of carbon, we might expect that the conversion of a fatty substance which is almost entirely composed of hydrogen and carbon, into water and carbonic acid, shall give off a far larger amount of heat than the combustion of a farinaceous or saccharine substance, in which there is only carbon to be burned off, the hydrogen being already united with oxygen in the proportion to form water. Experience shows that such is the case; for in the Arctic regions, ordinary bread is found to be very inefficient for the maintenance of animal heat, although an ample supply of oleaginous matters is completely effectual for this purpose; and, as Sir John Richardson has informed the Author, it has been recently found by the Hudson's Bay traders, that *maize* bread, which contains a considerable proportion of oil, is a most supporting food. Consequently, when the production of *fat* by the liver is suspended, and the production of *sugar* takes place, the amount of heat generated by the consumption of even an augmented quantity of the latter, will not be equal to that resulting from the combustion of the former; for only carbon will be burned off in the one case, whilst both carbon and hydrogen were consumed in the other.

We subjoin the general summary of the author at the end of this chapter, in which a synopsis of his views on several important points are presented:

We have now passed in review the chief among those components of the Human body—whether presenting themselves in its nutritive fluids, in its solid tissues, or in its excretory products—whose existence has been made definitely known by Chemists. It is by no means to be assumed that what has here been stated affords a complete list of the chemical components of this fabric ; still less, that the account which has been given of the metamorphoses they undergo is to be received in the light of a determinate scheme. We should, indeed, regard it as a mere sketch or outline, in which the broad features are conveyed with tolerable distinctness, but of which the details remain to be filled in by careful study. And the greatest advantage which can be derived from this method of viewing the subject, consists in the more definite boundary we are enabled to draw between what is known and what is unknown ; and again, between what rests on a fair basis of probability, and what has no better foundation than vague surmise. Moreover, there is an obvious advantage in combining the *chemical* and the *physiological* view of the facts in question. For the mere Chemist will not only be liable to continual error, when he attempts to reason as to what takes place in the *penetralia* of the living body from what he observes in his laboratory, without taking into account the difference in the conditions of the phenomena (of which kind of error some of the speculations of Prof. Liebig have afforded remarkable examples); but he will also be at a great disadvantage in the prosecution of his inquiries, for want of the guiding clue which physiological knowledge alone can afford. On the other hand, the Physiologist cannot safely make a step in advance, when engaged in the study of the metamorphoses of the alimentary materials into the living solids, and of the subsequent reduction of the latter to the condition of excrementitious matters, without relying on those exact data which Chemical tests and analyses can alone supply ; it being to these, in fact, that he owes whatever definite knowledge he possesses of the composition of these several bodies, with which it is of the most fundamental importance that he should be acquainted. Accordingly, all the most productive researches of recent times, in this department, have been the work of men, who have either combined within themselves these two kinds of knowledge, or who have brought them to bear upon one another from extraneous sources.

The following Summary is intended to convey, within a narrow compass, the leading conclusions, in regard to the Chemistry of the living Body, to which the Chemico-physiological labors of recent times appear to point.

I. The organic materials indispensable for the *genesis* of Tissue, consist of *Albuminous* and *Fatty* compounds. The former present themselves under various aspects, in the Vegetable as well as in the Animal kingdom, all being reducible, however, to the ordinary state of Albumen by the digestive process; and in their natural states of combination, they include most of the inorganic substances which are acquired in addition. There is no reason whatever to believe, that Albuminous compounds can be generated within the animal body, by the transformation of substances belonging to an entirely different type. The latter are directly afforded by ordinary animal food, and by many kinds of vegetable productions; and it seems to be when they are thus supplied, that they are most readily made available in histogenesis. They may be produced within the body, however, by the metamorphosis of either Albuminous or Saccharine compounds.

II. The great mass of those tissues of the body which belong to the *cellular* type, is generated at the expense of Albuminous matter; fat-particles, however, being intimately blended with this in an early stage of their formation. Of this we have a notable example in the case of Muscular tissue; but the cell-walls of all other textures would be found, if they could be entirely freed from their contents, to have the same composition. Even if they be altogether *chemically* identical, however, the molecular condition of the particles composing the amorphous coagulum and the living cell must be entirely different; and the latter exhibits distinctive *vital* properties, in virtue of the organizing process to which its material has been subjected. Not merely the cell-walls, but the cell-contents of these tissues (with the exception of those concerned in the act of excretion), seem to be derived from the Albuminous or from the Fatty constituents of the blood; this seems clear, for example, in regard to the globulin and hæmatin of the red corpuscles of the blood, and the horny matter of the epidermis and its appendages, which must have their source in the former; and also with respect to the contents of the adipose and nervous vesicles, which must be drawn wholly or in part from the latter. Whether, in the construction of the tissues of this class, the Albumen of the blood may serve directly as the *pabulum* for the production of cells, or whether it must needs pass first through the condition of Fibrin, cannot be distinctly affirmed; there is no positive evidence in support of either proposition; but the probabilities appear to the Author, on the whole, to favor the former view.

III. The great mass of the gelatigenous tissues of the body, whose texture is *simply fibrous*, is also derived from the albuminous element of the Blood; but this passes through the intermediate condition of Fibrin, which may be regarded as a substance endowed with the power of self-development into a low form of organized structure, and therefore as having already undergone a vitalizing influence. There is no sufficient reason to believe that Gelatin employed as food can ever be applied even to this purpose in the body; since all that we know of the *genesis* of the simple fibrous tissues indicates that, in assuming their characteristic structure, they pass through gradations similar to those which we witness in the production of the adventitious tissue of fibrinous exudations.

IV. When the death and disintegration of the tissues again bring their components under complete subjection to Chemical forces, an entirely different series of metamorphoses takes place, tending to degrade these components towards the condition of inorganic compounds. They would seem to resolve themselves into two classes of substances;—on the one hand, saccharine, oleaginous, and resinous matters, analogous to those of plants, in which carbon predominates;—on the other, a set of compounds peculiar to animals, of which nitrogen is the characteristic ingredient. From the albuminous constituent of muscle, for example, there is direct evidence that fat, sugar, and lactic acid may be generated on the one hand; on the other, creatine, and (probably through this creatine) urea, with the rest of the highly-azotized components of the urinary excretion. The sugar generated by the agency of the liver, from the products of the waste or disintegration of the system that are contained in the blood, seems to be at once employed in supporting the combustive process by which the animal heat is maintained. The fat may be directly applied to the same purpose, or may be stored up in the cells of Adipose tissue for future use. The peculiar resinous acids of the bile, which are probably formed from the same source, appear to fulfil, in part at least, a similar destination, after having been made subservient to other purposes. The lactic acid, chiefly generated in the substance of the muscles (probably by the metamorphosis of a saccharine compound, which may be looked on as the immediate product of their disintegration), is in like manner destined to be carried off by the respiratory process, though a part of it may first be rendered subservient to the digestive operation. But if the respiratory process should not be sufficiently active to remove these highly-carbonized compounds from the blood, we may find the lactic and hippuric acids in the urine, with the addition of carbonaceous pigmentary matter. On the other hand, the highly-azotized substances are destined for immediate elimination by the kidneys; their presence in the current of the circulation being so hurtful, that even a small amount of accumulation might induce fatal results.

V. Besides the foregoing substances, there are doubtless others which have not been so carefully studied, and which are passed off by distinct channels. Thus, we have no precise knowledge of those products of disintegration which are thrown off by the skin; and the proper fecal matter, which is undoubtedly derived from some excretion poured into the alimentary canal, rather than from putrescent changes in the residue of the substances which are passing through it, has not yet been made the subject of accurate examination.

VI. Where more alimentary matter is introduced into the blood than is required for the *genesis* of living tissue, this probably undergoes the same decomposing changes, as do the effete matters that are set free by the disintegration of the organized fabric. The saccharine and oleaginous matters are directly carried off by the combustive process, only that portion being applied to the production of adipose tissue which may not be required for the maintenance of the temperature of the body; whilst the albuminous and gelatinous appear to be resolved into the two classes of compounds already indicated, part of which are eliminated by the liver and lungs, the other part chiefly by the kidneys, but also by the skin and (its internal reflexion) the alimentary canal. It may here be mentioned, as an additional evidence of the production of sugar from albuminous compounds within the living body, that it is found in the milk of Carnivorous animals, which have been for some time restricted to a diet of animal flesh.

VII. The Inorganic acids, bases and saline compounds, which properly rank as constituents of the body, are for the most part applied to its construction in the forms in which they were introduced from the food; and they reäppear under the same forms in the excretions. But new compounds are also produced during the progress of the metamorphic changes already referred to. Thus, a portion of the sulphur taken in as a constituent of albuminous food, seems ty be oxidised in the final disintegration of the tissues by which that albumen was appropriated, and is converted into sulphuric acid; a part, however, still remaining unoxidized, and passing off in that state, both by the bile and the urine. So, again, if phosphorus (as such) be a constituent of the protien-compounds, or be united with fatty matters, it must undergo a similar oxidation within the system; as it scarcely ever presents itself in the excretions under any other form than that of phosphoric acid. On the other hand, by the oxidation of various organic acids largely contained in vegetable food, their alkaline bases are reduced to the state of carbonates, so as to be ready to combine with any of the stronger acids that may be present in the system; and ammonia seems also to be generated *de novo*. Thus a supply of bases is prepared, ready to neutralize not merely the acids whose mode of production has just been described, but also the uric, hippuric, and lactic acids which are generated within the body, and which do not readily pass off from it except in combination with bases; and according as the proportion of these bases is equivalent to that of the acids, exceeds it, or is exceeded by it, will the urine be neutral, alkaline, or acid.—When mineral substances, whose presence is superflous or noxious, are introduced into the body, an effort is usually made for their elimination, by some of the excretory organs; most commonly by the kidneys.

There is very strong evidence that, in all these transformations Chemical forces are alone concerned; this evidence arising, on the one hand, from the nature of the changes themselves; and, on the other, from the certainty that such forces *must* be in operation, and that their effects will be mod fied by the peculiar conditions under which they are exerted. We have already seen that many of the changes taking place within the living body can be precisely imitated out of it, by the use of means whose actual operation is of the same kind, though the *modus operandi* may be very different. Of this a remarkable example is afforded by the process of *oxidation*, which is continually going on within the system, and which produces a most important influence on the condition of the products of its disintegration. When the Chemist desires to convert one organic compound into another by oxidation, he treats it with some substance (*e. g.* nitric acid or peroxide of lead) which readily yields oxygen; whereas, the Physiological method is altogether different, for the substance to be acted on, being diffused through the circulating fluid is exposed to the direct influence of the oxygen of the air, in a state of almost infinitely minute division, during the passage of that fluid through the multitudinous capillary channels of the lungs. Now of the efficacy of this state of subdivision, in bringing about changes which do not occur when the substances to whose attractive forces these are due are simply exposed to each other *en masse*, we have a very remarkable example in the fact that iron, when reduced from the oxide to the metallic state by a current of hydrogen gas, and thus left as a very fine powder, becomes spontaneously ignited by exposure to common air, and oxidizes as rapidly as a piece of iron-wire would do when burned in pure oxygen or immersed in nitric acid. This point has scarcely been sufficiently attended to by Chemists, who have too readily satisfied themselves with accounting for such metamorphoses by laboratory operations, without inquiring how far similar agencies could be at work within the body: and we shall hereafter find that this powerful oxidizing process is probably employed, not merely in the combustion of materials introduced into the body, or supplied from itself, for the maintenance of its heat, nor only, in addition, for the removal of some of the products of its own disintegration, but also for the decomposition and elimination of certain organic poisons, from which, when they have been introduced into the body from without, it is freed through this channel, as it is from many of those of a mineral nature through the kidneys.—Allusion has already been made, again, to the peculiar action of

*ferments*, which tend to produce new and simpler arrangements of the elements of organic compounds, such as no ordinary reägents could effect. And it has been also pointed out that the very same substance in different stages of decomposition may occasion the genesis of several different sets of new compounds, according to the state in which it may itself happen to be when employed for this purpose. Moreover, it has been also seen, in how very marked a degree the condition and the metamorphoses of organic compounds are effected by the presence of very small quantities of inorganic substances; but whether their influence be that of ordinary chemical attraction, or that which has been termed "catalytic" power cannot yet be positively stated.—It is to be remembered, moreover, that the circulating fluid, which is probably the seat of most of the metamorphoses, is itself in a state of constant change; that new components are continually being introduced, on the one hand from the alimentary materials, and on the other from the disintegration of the tissues; and that such a condition must be eminently favorable to the chemical metamorphosis of its organic constituents.—It may be remarked, moreover, that the mode in which *capillarity* is brought into action in the processes of nutrition, is likely to have an important influence in determining further chemical changes, in virtue of the galvanic action which may thus be set up; it having been shown by Becquerel that, if the bend of a siphon be filled with fine sand, and an acid be poured into one leg, and an alkaline solution into the other, the chemical action which ensues at their point of meeting will give rise to a galvanic current between the contents of the two legs, when these are connected by a wire passing through the open ends of the siphon. As the blood and the tissues are continually acting chemically on one another through the permeable walls of the vessels, it is scarcely possible but that electric currents should be thus generated; and it seems very probable that these may perform an important part in the metamorphoses in question.

On the whole it may be confidently affirmed, that of the changes which have been described in the present chapter, there are none—save the production of Fibrin, whose peculiarly vital properties have been already dwelt on—which there is any adequate reason to attribute to other than Chemical agencies; for if all cannot yet be precisely imitated by laboratory processes, this imitation has been successfully practised in the case of a large number of them; and the nature of the remainder is such as leaves it by no means improbable that they too will be reduced to the same category. In treating of this subject, it would be very easy to obtain a temporary solution of all difficulties of this kind, by regarding every case not otherwise explicable as a manifestation of "vital force;" but such a method is altogether inconsistent with sound philosophy; and we have no right to call in the assistance of vital force on any other occasion than when we witness phenomena which are not only inexplicable by, but altogether inconsistent with, the known operations of Physical and Chemical forces. Phenomena of this kind will hereafter come under our consideration; but none such (with the single exceptivn just referred to) have yet fallen under our notice; the metamorphoses we have been considering being mere changes in composition, analogous to those which *have* been effected in the laboratory; and the elements alike of the original substances, and of the products of their changes, being held together by affinities in which Life has obviously no concern.—It is not here denied, however, that Vital Force has an influence upon the Chemical phenomena of the body; on the contrary, much evidence will be hereafter given, that such an influence *is* exerted, especially through the nervous system. But this agency may be considered to operate, after the manner of Electricity or Heat, in modifying the play of ordinary Chemical attractions, rather than in substituting for them a new set of "vital affinities."

The highest estimate, however, that we seem justified in making, of the play of Chemical Affinities in the living body, is applicable only to those transformations, which, on the one hand, prepare the *pabulum* for that Orgnizing process, whereon is dependent the development of *vital* properties in substances that were previously inert; and which, on the other, minister to the application of the effete matters, resulting from the disintegration of tissues whose term of life is over, to purposes that are advantageous to the system in general, or are subservient to their removal in the most effective manner from the economy, to whose operations their continued presence would be prejudicial. Thus the reduction of all protein compounds to the condition of Albumen, in the digestive process, and the introduction of this substance into the blood in its soluble form, can be counted nothing else than a purely chemical operation. The same may be said of the conversion of starch into sugar, and of sugar into fatty matter. It may be surmised, moreover, that the production of globulin and of hæmatin from albuminous material, may be due to chemical actions determined by the vital agency of the floating cells of the blood, in the manner indicated in the preceding paragraph. And the same may be true of the change of composition (if any difference really exists), which is a part of the metamorphosis of albumen into fibrin; and of that still more decided change which subsequently takes place, when the fibrous tissues are converted, in the act of formation, into the substance which yields gelatin. For, as already pointed out, although the Chemist has not yet succeeded in imitating this metamorphosis, yet there are circumstances which indicate that such a fundamental relation exists between the protien-compounds and gelatin as leaves no right to assume that any other than chemical forces are concerned in it.

But with the changes directly concerned in the *development of living tissue*, Chemistry would seem to have nothing whatever to do. The substance of Muscle, for example is *chemically* the same with the Albumen of the blood; and the whole difference between the organized structure possessed by the former, and the amorphous coagulum formed by the latter, between the marvelous activity of the one and the passive inertness of the other, must be attributed to Vital force alone. Now it is one of the chief peculiarities of this Vital force, that it is able, so long as it is capable of being fully exerted, to resist and keep at bay the influence of those Chemical and Physical forces, which would tend, were it not for this property of the living substance, to effect its speedy disintegration and decay. Of this we have a most characteristic and apposite example, in the case of a seed that has been brought to the surface of the soil, after having been buried for a long lapse of years or centuries in the earth. Whilst it remained in complete seclusion from moisture and oxygen, and was kept at a low temperature, no appreciable alteration took place in it; but so soon as it is exposed to warmth, and to the contact of air and water, it *must* begin to change—its passive existence giving place to a state either of growth or of decay, according as it has retained, or has lost, its vital properties. For the very agents which are most effectual in stimulating it to vital activity, and which afford the conditions, dynamical and material, whereby the seed develops itself into the plant, are those which, if the seed be no longer capable of germination, most favor its decay, reducing its organic components back to the condition of inorganic compounds. This peculiar attribute of living substances will be more fully considered in the next Chapter.

Immediately, however, that we pass the confines of Life, we reënter the domain of Chemistry; for so soon as the vital activity of the living tissues has ceased, their materials again become entirely subject to Chemical forces; and all the metamorphoses which we have been occupied in tracing out—tending, as they do, to reduce the organic compounds with which they commence, lower and lower in the scale, until these are restored to the forms of simple binary composition from which their elements were at first derived—can scarcely be regarded in any other light than as the result of ordinary Chemical agencies. Thus, then, according to the view here advocated, the Life of each part is dependent upon Chemical operations, in so far as it is by these that its nutrient materials are prepared, and the products of its decomposition are carried away; but the application which it makes of such materials to the production of new organized tissue, and the various actions which that tissue then exerts in virtue of its organization, are not only incapable of being explained on Chemical principles, but often take place in direct antagonism to Chemical forces.

